# Prednisolone exerts exquisite inhibitory properties on platelet functions

**DOI:** 10.1016/j.bcp.2012.02.006

**Published:** 2012-05-15

**Authors:** Elisabetta Liverani, Sreemoti Banerjee, Wayne Roberts, Khalid M. Naseem, Mauro Perretti

**Affiliations:** a William Harvey Research Institute, Barts and The London School of Medicine, Queen Mary University of London, London, United Kingdom; b Centre for Cardiovascular and Metabolic Research, Hull York Medical School, University of Hull, Hull, United Kingdom

**Keywords:** Platelet biology, Prednisolone, Glucocorticoid receptor, Adhesion, Aggregation, Cell-to-cell interaction

## Abstract

We have previously reported presence of the glucocorticoid (GC) receptor (GR) alpha on blood platelets, and its ability to modulate platelet aggregation when activated by the synthetic GC prednisolone (Pred). In the present study we investigated the effects of Pred on broader aspects of platelet functions to unveil novel non-genomic actions on this cell type. Using whole blood assay we demonstrated that Pred was the only GC able to inhibit platelet aggregation and platelet–monocyte interactions. This latter effect was due to regulation of platelets, not monocytes. We next examined the effects of Pred on physiological actions of platelets, observing inhibition of platelet adhesion and spreading on collagen under static conditions. Moreover Pred inhibited thrombus formation under flow, suggesting potential important effects in haemostasis and thrombosis. Pred was unable to regulate platelet reactivity under conditions where the effects of platelet-derived ADP and TxA_2_ were blocked, suggesting that the GC targeted the activation-dependent component of the adhesion and aggregation response. The effects of Pred were not mediated through cyclic nucleotide signaling, but rather seemed to evolve around selective regulation of P2Y_12_ ADP receptor signaling, intimating a novel mode of action. This study details the actions of Pred on platelets unveiling novel properties which could be relevant for this GC in controlling unwanted vascular and thrombotic diseases.

## Graphical abstract

**Figure:**
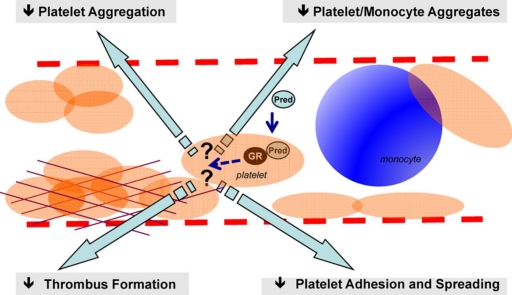

## 1 Introduction

In virtually all cell types, glucocorticoids (GC), lipophilic compounds with rapid diffusion through the plasma membranes, bind to cytosolic glucocorticoid receptors (GR) to form a receptor complex which acts as a transcription factor affording remarkable modulation of gene expression [1,2]. The GC/GR complex can exist either as a monomer or a dimer; while the monomer will remain in the cytosol, affecting signaling cascades and gene promoter activity indirectly, the dimer translocates into the nucleus, binds to the GC response elements present in promoter regions of target genes, hence modulating gene expression directly. Alongside these classical GR responses, which are in line with those of other sex hormone receptors, more rapid effects have been recently reported for GC, classified as “non-genomic”, since they do not involved genetic modification [3]. The detailed mechanisms of GR activation by GCs to elicit “non-genomic” effects are still unclear, though some hypothesis have been put forward [4]: GC can affect cell responsiveness by accumulating into plasma membranes changing cell physic-chemical properties [5]. Furthermore, selective membrane effects can be elicited in view of the existence of membrane receptor sites on monocytes [6]. However, GC binding to classical GR can also initiate rapid non-genomic responses, including modulation of T cell receptor-mediated phospho-signalosome [7,8] and annexin A1 phosphorylation and secretion in neutrophils [9].

Our previous work has revealed the unexpected finding that GR activation can modulate platelet function [10]. These cells play a key role in haemostasis and thrombosis and, being anucleate cells, they represent the ideal model to study the non-genomic effects of GC. Indeed, we reported that prednisolone (Pred), but not dexamethasone (Dex), inhibits platelet aggregation *in vitro* [11]. Since most laboratories use Dex as a reference GC for *in vitro* studies, this differential efficacy would provide an explanation why the effects of GC on human platelets have been neglected for so long. Of relevance, the inhibitory effect of Pred was reversed by pre-incubation with the GR antagonist mifepristone, suggesting the functional importance of the classical GR/GC complex also in the inhibition of platelet aggregation in platelet-rich plasma [11].

Blood platelets play an established role in the thrombotic complications arising from atheroma-induced vascular rupture, and they can also contribute to the inflammatory responses leading to endothelial dysfunction [12]. Thus the potential regulation of platelet function by therapeutic GC such as Pred provides an interesting addition to their clinical usage. In the present study we set out to characterize the effects of Pred in human platelet functions, spanning from monitoring aggregation, using a whole blood protocol, to establishing the effect of this GC on other responses of activated platelets, including formation of platelet/monocyte aggregates and platelet adhesion under arterial flow. Our data demonstrate that in whole blood Pred, but not other GCs, modulate platelet-platelet interactions, platelet–monocyte interaction and thrombosis under conditions of flow in a GR-dependent manner. Collectively, these results shed novel light on the biology of Pred in relation to the human platelet and, in view of its widespread application for the management of several clinical conditions, may have bearing to human pathology.

## 2 Materials and methods

Unless otherwise specified, all chemicals were from Sigma–Aldrich (Poole, UK).

### 2.1 Blood collection and platelet isolation

Human blood was collected from healthy volunteers, who had abstained from non-steroidal anti-inflammatory drugs medications at least for the preceding 7 days, using venepuncture into hirudin (1:9, Instrumentation laboratory, Warrington, UK). All experiments were approved by the East London Research Ethics Committee (no. 05/Q0603/34).

For preparation of washed platelets, blood was taken into acid citrate dextrose (ACD: 29.9 mM sodium citrate, 113.8 mM glucose, 72.6 mM sodium chloride and 2.9 mM citric acid, pH 6.4) as anticoagulant. Platelet-rich plasma (PRP) was obtained by centrifugation of whole blood at 200 g at 20 °C for 20 min. PRP was treated with citric acid (0.3 mM) and centrifuged at 1900 rpm for 12 min. The platelet pellet was then suspended in wash buffer (36 mM citric acid, 10 mM EDTA, 5 mM glucose, 5 mM KCl, 9 mM NaCl) and spun once more. Platelets were finally re-suspended at a concentration of 5 × 10^7^ or 2.5 × 10^8^ platelets/ml in modified Tyrodes buffer (150 mM NaCl, 5 mM HEPES, 0.55 mM NaH_2_PO_4_, 7 mM NaHCO_3_, 2.7 mM KCl, 0.5 mM MgCl_2_, 5.6 mM glucose) unless otherwise stated.

### 2.2 Platelet aggregation

Whole blood impedance aggregometry was applied using a Multiplate™ analyzer (Instrumentation Laboratory, Munich, Germany). Thirty minutes after venepuncture, 300 μl of hirudinated blood was diluted 1:1 with saline (0.9% w/v, Sigma–Aldrich) into a test cell at 37 °C and stirred for 3 min in presence of one or combination of GCs; prednisolone (Pred), dexamethasone (Dex), nitro-dexamethasone (Nitro-Dex), fludrocortisone (Fludro), triamcinolone (Triam) before the addition of either collagen (3.2 μg/ml; Instrumentation Laboratory), arachidonic acid (AA, tested at 0.5 and 0.25 mM; Instrumentation Laboratory) or adenosine-diphosphate (ADP, tested at 3.2 and 6.5 μM; Instrumentation Laboratory). The electrical resistance between two pairs of silver coated wires was measured and plotted over a 6-min period. The Multiplate™ analyzer calculated the average area under the curve (AUC) for the two readings. Each test was conducted in duplicate for each donor sample.

Alternatively, washed platelets (250 μl; 2.5 × 10^8^ platelets/ml) were incubated for 5 min at 37 °C in a multichannel Chronolog Aggregometer (Pennsylvania, USA) prior to stimulation to allow for temperature equilibration. The platelet suspension was then stimulated with ADP (5 μM) under stirring conditions (1000 rpm) and platelet aggregation was recorded for 4 min. The platelet aggregometer was recalibrated for every individual platelet sample used. The following incubation times were used for inhibitors added prior to stimulation of platelets; A3P5P (300 μM for 1 min), MRS2395 (10 μM for 1 min) and Pred (1 or 10 μM for 5 min) prior to the addition of ADP (10 μM).

### 2.3 Platelet–monocyte aggregation assay

The formation of platelet–leukocyte aggregates in the whole blood was analyzed by pre-incubating 100 μl of blood with antibodies (diluted 1:50) against human CD42b (FITC-conjugated; clone HIP1; eBioscience, Hatfield, UK) and CD14 (PE-conjugated; clone 61D3, eBioscience, Hatfield, UK) and either Pred or vehicle for 3 min at 25 °C. Platelets were stimulated by adding ADP (1–3 μM) and incubated for a further 20 min at 25 °C. Samples were fixed and red blood cells (RBC) lysed using BD FACS™ lysing solution (BD Bioscience; 1:10 final dilution) and kept at 4 °C up to analysis.

In some cases, Pred was added to either the platelet or the leukocyte preparation separately. To this end, after removing PRP, the remaining of the blood was resuspended in RPMI 1640 (BioWhittaker, Wokingham, UK) (ratio 1:1) and mononuclear cells separated through a double density gradient as previously described [17]. Both PRP and mononuclear cells pretreated with either Pred (5 nM–5 μM) or vehicle for 5 min at 25 °C. Following a washing step, platelets and mononuclear cells were re-suspended in Hepes buffer (NaCl 150 mM, KCl 5 mM, MgSO_4_ 1 mM, HEPES 10 mM) and added to tubes in equal volume. After addition of FITC-conjugated anti-human CD42b and PE-conjugated anti-human CD14, samples were stimulated with ADP (1–3 μM) and incubated for 20 min at 25 °C then fixed using paraformaldyde (4%, Sigma–Aldrich, Poole, UK) and stored at 4 °C prior analysis. Flow cytometry was performed on a FacsCalibur analyzer (Becton Dickinson, Oxford, UK). Platelets and monocytes were discriminated by forward and side light scatter, identified by their positive staining for FITC-CD42b or PE-CD14, respectively. Events double positive for FITC and PE identified platelet–monocyte aggregates and were recorded as a percentage of a total of 10,000 gated monocytes.

### 2.4 Fluorescence microscopy

Platelets (5 × 10^7^/ml) the presence and absence of the appropriate inhibitors were adhered to collagen (10 μg/ml)-coated coverslips for 30 min at 37 °C. After incubation, coverslips were washed with PBS to remove non-adherent platelets. For experiments with Pred, the GC was added to platelets 5 min before addition to slides. The following incubation times were used for inhibitors added prior to adhesion of platelets: apyrase (2 U/ml for 1 min), indomethacin (10 μM for 5 min), ODQ (20 μM for 15 min) or myrPKI (500 nM for 15 min). Adherent platelets were fixed with 3.5% paraformaldehyde and permeablized with 0.2% Triton-X-100 in PBS. Platelets were stained for F-actin using TRITC-conjugated phalloidin and visualized using fluorescent microscopy (IX71, Olympus, Kyoto, Japan). For each experiment, the numbers of platelets from eight random fields of view were added, and the results were calculated as mean number of adherent platelet per 0.01 mm^2^. The average surface area of the individual adherent platelets was calculated using Cell P software (Olympus).

### 2.5 Analysis of protein phosphorylation in platelets

Washed platelets (2 × 10^8^ platelets/ml) were pre-treated with either ODQ (20 μM) or myrPKI (500 nM) for 10 min and then incubated with either the nitric oxide donor, S-nitrosoglutathione (GSNO; 10 μM) or prostaglandin E1 (1 μM) for 2 min. Platelets were lysed by the addition of Laemmli sample buffer (1×). Proteins were separated by sodium dodecylsulfate polyacryl-amide gel electrophoresis (SDS-PAGE) (7.5% polyacrylamide gels) and immunoblotted as previously described [13]. Membranes were with either anti-VASP-phospho^239^ or anti-VASP-phospho^157^ antibodies (1:1000).

### 2.6 Platelet aggregation under flow

Platelets (2 × 10^8^ platelets/ml) were incubated with dihexloxacarbocyanine (DIOC_6_; 1 μM) at 37 °C for 10 min and then incubated at 37 °C for 2 min with Pred (10 μM), before being reconstituted with autologous washed RBC. Flow studies were performed using glass capillary tubes (Camlab; Cambridge, UK), coated with immobilized collagen (100 μg/ml; Nycomed, Zurich, Switzerland) for 12 h, blocked with BSA/PBS (5 mg/ml) for 1 h and washed under flow conditions with PBS for 4 min. Platelets were perfused through collagen-coated capillary tubes at 1000/s^−1^ for 4 min, followed by washing (4 min) at the same shear rate. Adherent platelets/thrombus formation was visualized using fluorescence microscopy (IX71, Olympus). Data were calculated from the percentage of the area covered by adhering platelets in a defined area (surface coverage), since this methodology cannot fully discriminate between individual platelets and platelet aggregates [14]. For each experiment 10 random fields of view used to calculate surface coverage (%) using ImageJ software (NIH).

### 2.7 Statistics

Experiments were conducted in duplicate or triplicate and repeated at least 3 times. Data are reported as mean ± SEM. Statistical differences were analyzed by ANOVA followed by Dunnett's test or by Student's *t* test, as appropriate. A *P* value less than 0.05 was taken as significant.

## 3 Results

### 3.1 Prednisolone inhibits platelet function in whole blood

**Fig. 1:**
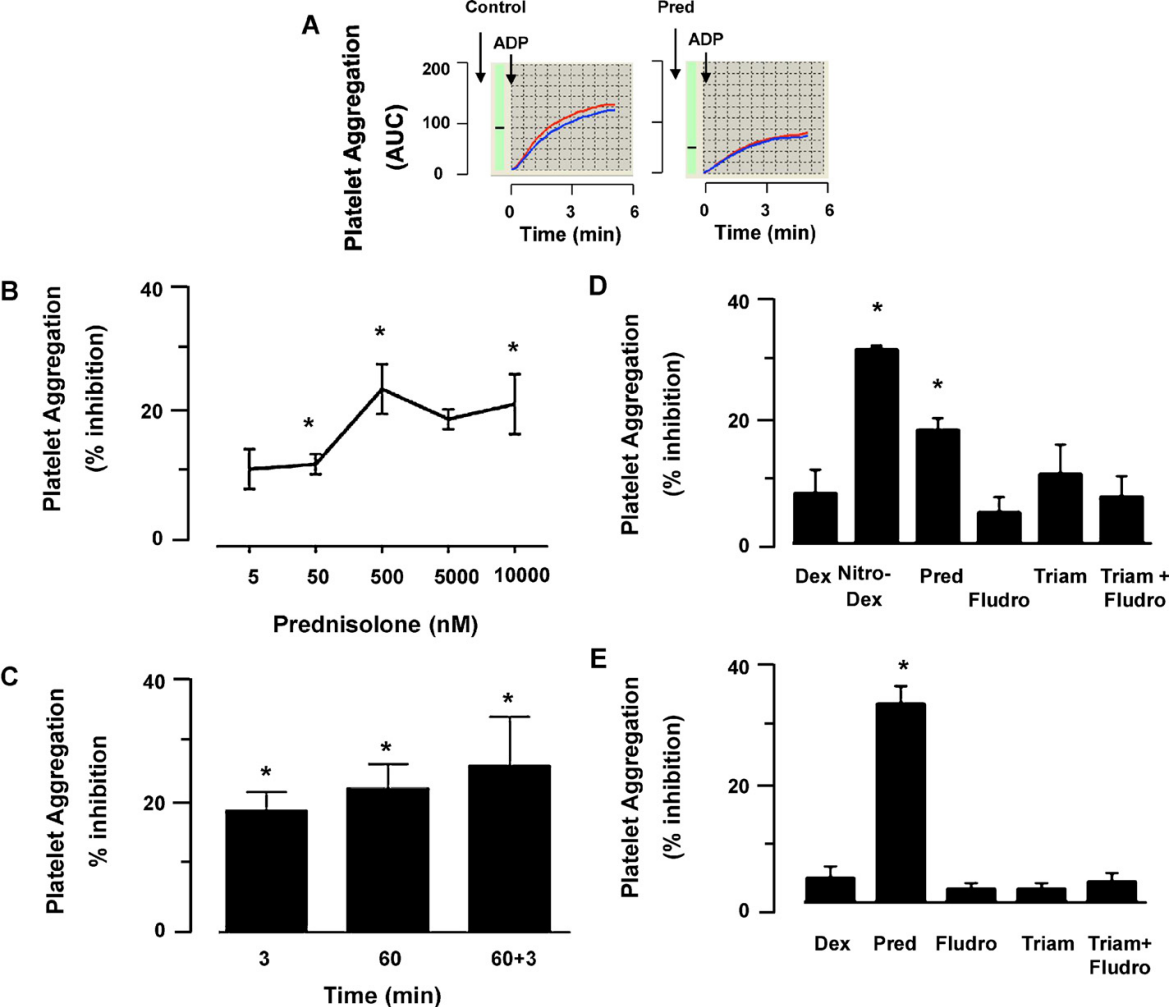
Prednisolone inhibits ADP-induced platelet aggregation in whole blood. (A) Representative platelet aggregation curves as determined with the whole blood aggregometer, allowing quantification of the area under the curve (AUC). Curves are from ADP (3.25 μM) with or without Pred (10 μM), showing duplicate readings. (B) Concentration–response curve for Pred against ADP (3.25 μM) as stimulus; *n* = 5, *P < 0.05 compared with untreated samples. (C) Lack of tachyfilaxis to Pred effect. The GC was incubated with the blood for 3 min, 60 min or 3 + 60 min (added twice) prior to ADP-induced aggregation. Data, mean ± SEM of 3 experiments, are reported as % of inhibition as compared to appropriate control samples, were vehicle was added; *P < 0.05 compared to control. (D) The reported GC were tested in the whole blood assay, using a 3 min pre-incubation period and ADP (3.25 μM) as stimulus. Prednisolone (Pred, 10 μM), dexamethasone (Dex, 10 μM), Nitro-dexamethasone (Nitro-Dex, 10 μM), triamcinolone (Triam, 10 μM), fludrocortisol (Fludro, 10 μM) and a combination of Fludro and Triam (5 μM each); data are mean ± SEM of 5 distinct experiments; *P < 0.05 compared to vehicle. (E) As in (D) except experiments were conducted with platelet rich plasma (PRP). Data are mean ± SEM of 4 distinct experiments; *P < 0.05 compared to vehicle.

Incubation of whole blood with Pred (5 nM–10 μM) led to a concentration-dependent inhibition of ADP-induced platelet aggregation (Fig. 1A and B). Modest inhibitory effects were observed with concentrations as low as 5 nM, with optimal inhibition of aggregation observed at 500 nM, when aggregation was reduced by 22 ± 4% (*n* = 5). Increasing the concentration to supra-physiological levels such as 10 μM failed to augment the extent of inhibition afforded by Pred. In the same settings, aspirin (20 mg/ml) produced 70% inhibition of ADP platelet aggregation (not shown). In any case, the inhibitory effects of Pred were rapid, being established within 3 min of incubation and remained consistent for up to 60 min (longest time tested) post-steroid application (Fig. 1C). There were no signs of tachyfilaxis, so the double addition of Pred at both 3 and 60 min prior to ADP addition, did not lead to a reduced extent of inhibition (Fig. 1C). In the whole blood assay, Pred (tested at 10 μM) failed to significantly attenuate platelet aggregation elicited by arachidonate or collagen (Table 1).

Next we aimed to determine whether these inhibitory properties of Pred were shared by other GCs and therefore compared its anti-platelet actions with those of Dex, Nitro-Dex, fludrocortisone, triamcinolone and a combination of the latter two. Consistent with previous experiments Pred inhibited aggregation in whole blood (19 ± 3%; *P* < 0.05). Nitro-Dex also caused a significant inhibition of aggregation (29 ± 7%; *P* < 0.05), although this is likely to be related to the NO-donor properties since the parent compound Dex alone had no effect. Fludrocortisone and triamcinolone were also ineffective platelet regulators (Fig. 1D). Similar results were obtained when the same GCs were tested in PRP, confirming the unique key property of Pred (Fig. 1E).

### 3.2 Pred modulates platelet–monocyte interactions in whole blood

**Fig. 2:**
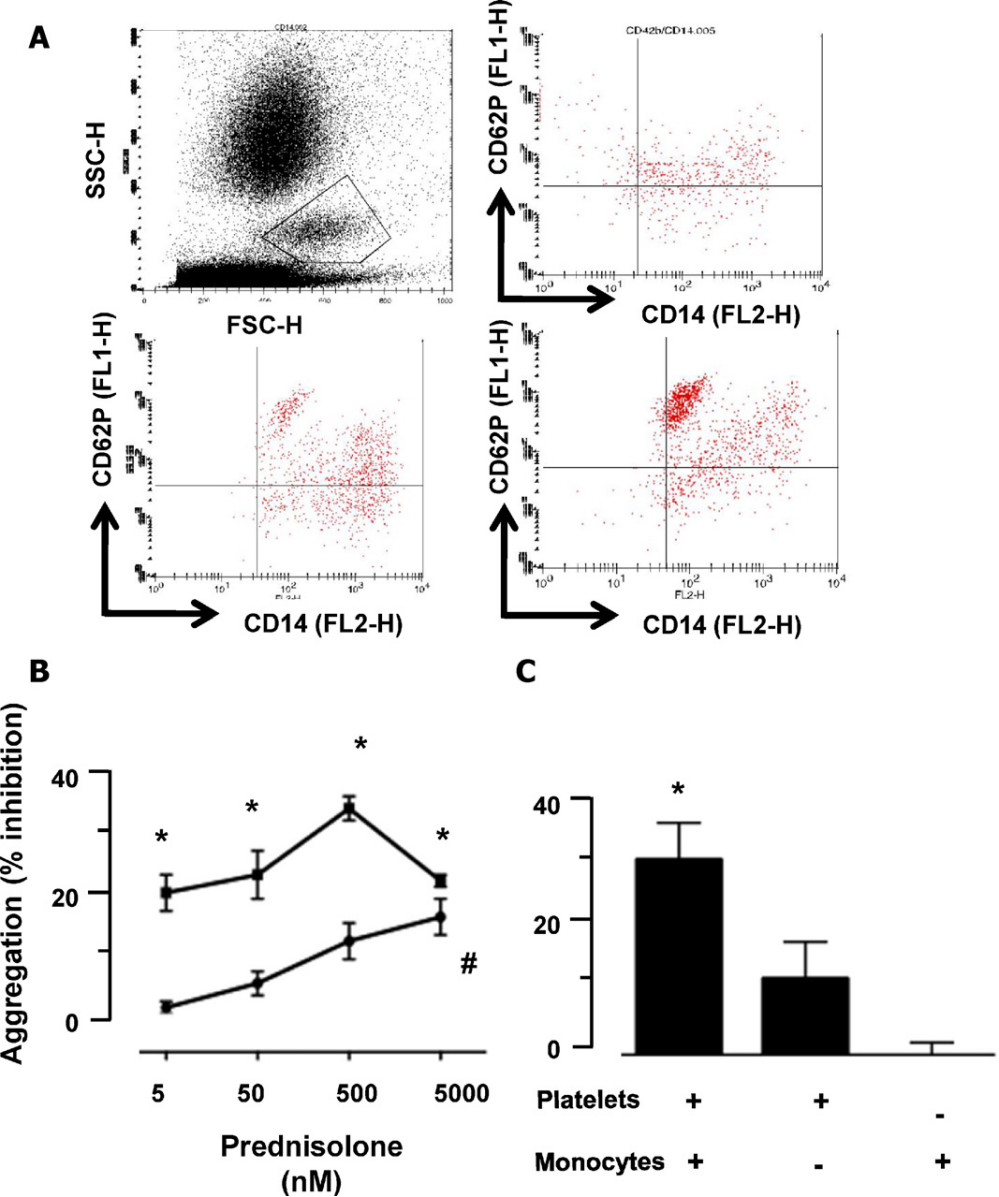
Prednisolone preferentially targets the platelet to inhibit platelet/monocyte aggregate formation. Formation of platelet/monocyte aggregates was investigated using flow cytometry. (A) Top let panel: scatter plot indicating the monocyte population (gate). Top right panel, plot for CD42b and CD14 in unstimulated identifies the quadrant for double positive events. Bottom right, blot after ADP (3.25 μM) addition. Bottom left, Pred (5 μM) was incubated for 5 min with the blood prior to ADP addition: note the lower extent of double positive events. (B) Concentration response for Pred inhibition of aggregates formation upon stimulation with 1.56 (closed squares) or 3.25 (closed triangles) μM ADP. Data are mean ± SEM of 5 experiments performed in triplicate; *P < 0.05 compared to vehicle. (C) Identification of the platelet as the main target for Pred. Monocytes and platelets were exposed, contemporarily or separately, to 500 nM Pred for 3 min prior to reconstitution and stimulation with 1.56 μM ADP. Data are mean ± SEM of 5 experiments performed in triplicate; *P < 0.05 compared to vehicle.

To determine the breath of Pred effects on platelet functions, beyond aggregation [11], a flow cytometry protocol was applied to examine platelet–monocyte aggregate formation. Addition of ADP (1 and 3 μM) to whole blood produced a clear increase in platelet–monocyte interactions (Fig. 2A), with 28 ± 5 and 45 ± 4% aggregate formation, respectively (compared to background). Pre-incubation of whole blood with Pred (from 5 nM to 5 μM; 3 min) prior to ADP led to a concentration-dependent inhibition of platelet/monocyte aggregates. For instance, at 5 nM Pred caused 20 ± 4% inhibition of ADP (used at 1 μM) induced aggregates formation, while maximal effects (32 ± 2%; *P* < 0.05) were observed with the 500 nM concentration. This GC retained its inhibitory effect also when a higher concentration of ADP (3 μM) was used, though the highest concentration tested of 5 μM was required to attain significant inhibition (Fig. 2B).

**Fig. 3:**
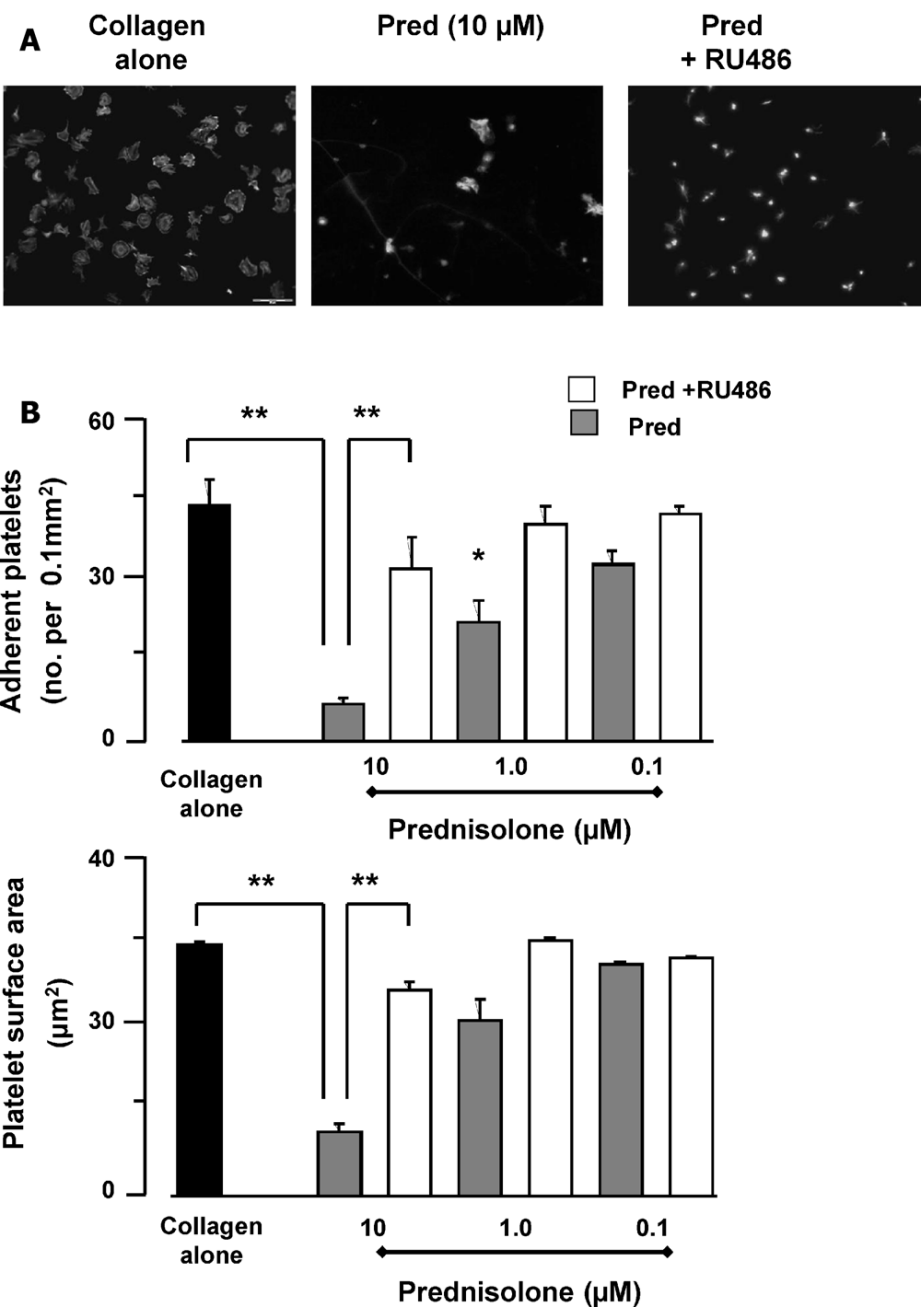
Prednisolone potently inhibits platelet adhesion and spreading on collagen. Glass slides were coated overnight at 4 °C with collagen (100 μg/ml), or heat-denatured humans serum. Uncoated surfaces were blocked by overlay with heat inactivated human serum albumin (HSA, 5%) for 30 min at 20 °C. Washed platelets (5 × 10^7^ platelets/ml) were pre-incubated with Pred (5 min) (0.1, 1 and 10 μM) in the presence and absence of RU486 and adhered for 30 min, stained with TRITC-phalloidin and viewed using the 60× magnification. (A) Representative images. Bar, 20 μm. (B) Platelets from 8 random visual fields with a total area of 0.1 mm^2^ were counted and expressed as number of adherent platelets/0.1 mm^2^. (C) These images were used to determine platelet surface area, presented the mean surface area (μm^2^) ± SEM. All data shown are mean ± SEM, *n* = 5 with separate blood donors. *P < 0.05 compared to collagen alone.

To clarify if one of the two cell types selected was a preferable target for the GC, we treated PRP and monocytes separately with Pred (500 nM), before reconstituting the co-culture system and stimulating with ADP (1 μM). When both cell types were treated with Pred, we observed a 30 ± 6% inhibition of aggregate formation (*P* < 0.05) (Fig. 2C). When platelets alone were exposed to the GC, its ability to inhibit platelet–monocyte aggregates was still apparent albeit slightly reduced (12 ± 4% reduction). However, if the monocytes were selectively treated with Pred, no inhibition of cell aggregates could be observed (Fig. 3C). Taken together with the platelet-to-platelet aggregation data, these results identify platelets as a primary cellular target for Pred, and they also underline that the properties of this GC are maintained even in the presence of other cells, increasing its physiological and pharmacological relevance.

### 3.3 Prednisolone inhibits platelet adhesion and spreading on immobilized collagen

Having established that Pred inhibits platelet aggregation, a response that relies on integrin-mediated platelet-platelet interaction [15], we next explored whether the GC could also affect platelet-extracellular matrix interactions by examining platelet adhesion to collagen. Using fluorescence microscopy, we observed that incubation of washed platelets with collagen-coated coverslips led to a marked degree of cell adhesion (43.2 ± 4.7 platelets/0.01 mm^2^). When platelets were pre-incubated with Pred a concentration dependent inhibition of adhesion was observed. Threshold effects were observed with 1 μM Pred, with a number of adherent platelet reduced to 21.8 ± 4 platelets/0.01 mm^2^, while maximal effects occurred at 10 μM Pred, when adherent platelets fell to 6.8 ± 2.9 (hence > 80% reduction; *P* < 0.01) (Fig. 3B). In addition to reducing the number of adherent platelets, Pred also inhibited the ability of platelets to spread on collagen (measured as surface area). At 10 μM Pred, platelet spreading was reduced from 29.5 ± 1 in the absence of Pred to 2.3 ± 1 μm^2^ (*P* < 0.05) in the presence of values Pred. The inhibitory effects of Pred (10 μM) on platelet adhesion to collagen were reversed by the GR antagonist, RU486 (10 μM; Fig. 3C). Similarly, where Pred (10 μM) reduced platelet spreading from 29.5 ± 1 to 2.3 ± 1 μm^2^, the mean surface area remained at 24.1 ± 1.8 μm^2^ in the presence of RU486. Similar data were obtained using fibrinogen as the adhesive surface (Supplementary Fig. 1). Of interest, we tested Dex (10 μM) in this assay and it resulted inactive (data not shown), in line with the results obtained with the experiments of whole blood aggregation.

### 3.4 Pred inhibits platelet adhesion by targeting secondary mediator induced platelet activation

**Fig. 4:**
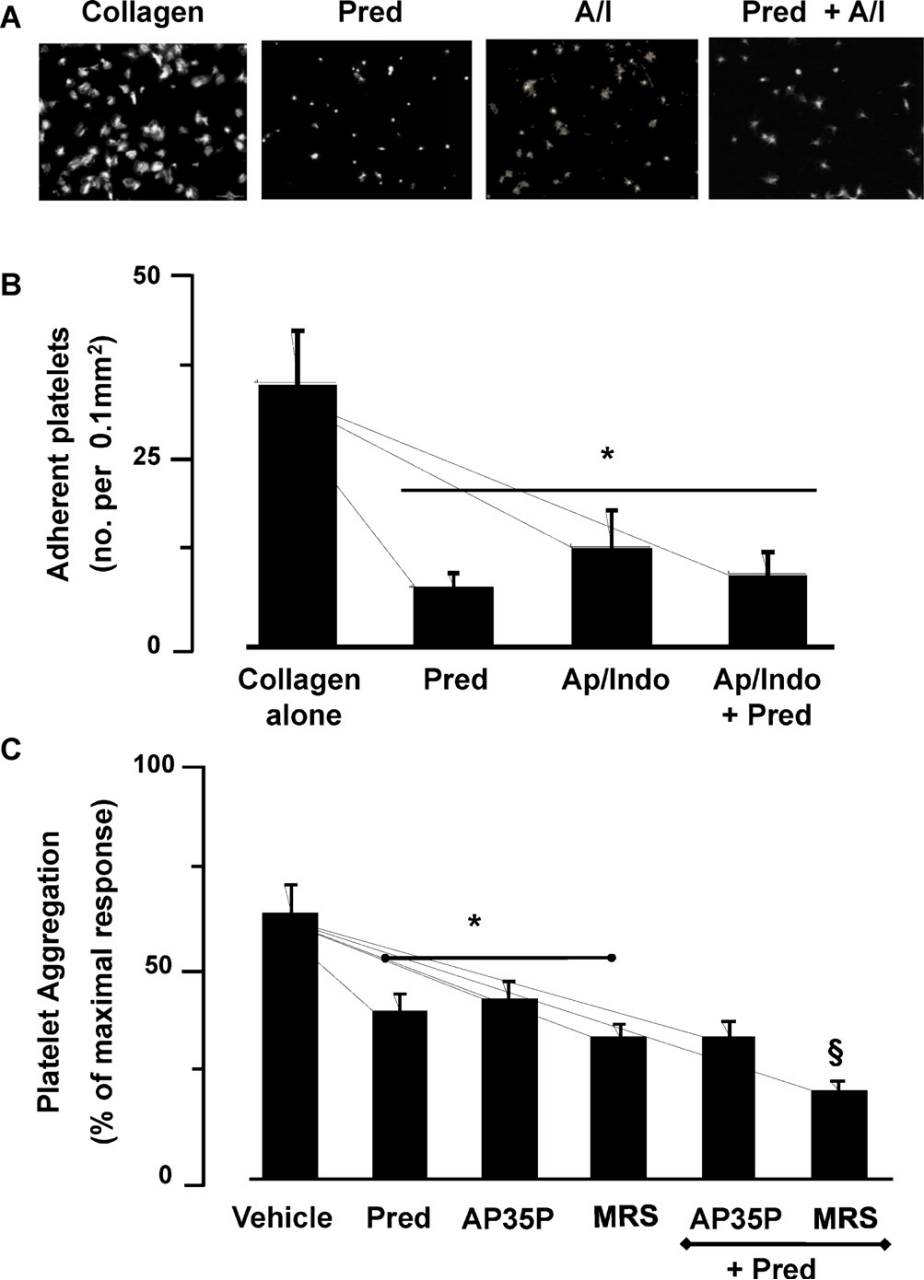
Prednisolone effect on platelet adhesion under conditions that abrogate ADP and TxA_2_ activity. Glass slides were coated overnight at 4 °C with collagen (100 μg/ml), or heat-denatured humans serum. Uncoated surfaces were blocked by overlay with heat inactivated human serum albumin (HSA, 5%) for 30 min at 20 °C. Washed platelets (5 × 10^7^ platelets/ml) were pre-incubated with Pred (5 min; 10 μM) in the presence and absence of apyrase (Ap; 2 U/ml) and indomethacin (Indo; 10 μM), and then adhered for 30 min, stained with TRITC-phalloidin and viewed using the 60× magnification. (A) Representative images. Bar, 20 μm. (B) Platelets from 8 random visual fields with a total area of 0.1 mm^2^ were counted and expressed as number of adherent platelets/0.1 mm^2^. All data shown are mean ± SEM, *n* = 4 with separate blood donors. *P < 0.05 compared to collagen alone. (C) Washed platelets (2.5 × 10^8^ platelets/ml) pre-incubated with Pred (1 μM) and then stimulated with ADP (5 μM) alone or in the presence of A3P5P (300 μM) or MRS2395 (MRS; 10 μM), and the aggregation response was monitored for 4 min under constant stirring. All data shown are mean ± SEM, *n* = 4 with separate blood donors. *P < 0.05 compared to ADP in the presence individual inhibitors, ^§^*P* < 0.05 compared aggregation with either Pred or MRS2395 alone.

Having established the ability of Pred to regulate a number of platelet functions, we began to examine the potential mechanisms. We have shown that platelet adhesion to collagen requires an activation-dependent cognate response, driven through platelet derived ADP and TxA_2_ [13]. Hence, we investigated the role of these soluble agonists in the effects of Pred. The joint presence of apyrase (1 U/ml) and indomethacin (10 μM), reduced platelet adhesion by 36 ± 4 platelets/0.01 to 12.3 ± 7.2 mm^2^ (60% inhibition; *P* < 0.05), confirming that maximal adhesion requires substantial platelet activation driven by platelet-derived metabolites (Fig. 4B). Pred (10 μM) reduced the number of adhered platelets to 9.0 ± 3.0 platelets per 0.01 mm^2^ (Fig. 4). When apyrase/indomethacin-treated platelets were incubated with Pred, 9.4 ± 3.0 mm^2^ platelets adhered to collagen, which was not significantly different to that attained by the combination apyrase/indomethacin alone. Thus, collagen-induced adhesion and spreading in the presence of apyrase/indomethacin is unaffected by Pred, indicating that the inhibitory actions of the GC are targeting the secondary mediator-induced activation-dependent component of the platelet adhesion process.

To further understand how Pred was regulating the actions of secondary mediators, we examined whether individual ADP receptors were being targeted. In order to simplify the experimental approach, we examined aggregation of washed platelets stimulated with ADP (5 μM) in the presence of MRS2395 (10 μM) and A3P5P (300 μM) to inhibit the potential effects of ADP signaling through P2Y_1_ and P2Y_12_ receptors respectively. Under these conditions Pred reduced aggregation from 68 ± 4 to 43 ± 7 (*P* < 0.05), while A3P5P and MRS2395 reduced aggregation to 46 ± 8 and 36 ± 7% respectively (*P* < 0.05). Interestingly when platelets were treated with prednisolone in combination with A3P5P, aggregation was reduced to 36 ± 5%, which was no different to when the inhibitors were used independently. In contrast, a combination of prednisolone with MRS2395 reduced aggregation to 22 ± 3% (*P* < 0.05 when compared to either inhibitor used alone) (Fig. 4C). These data suggest that prednisolone may have the capacity to target P2Y_12_/Gi signaling.

**Fig. 5:**
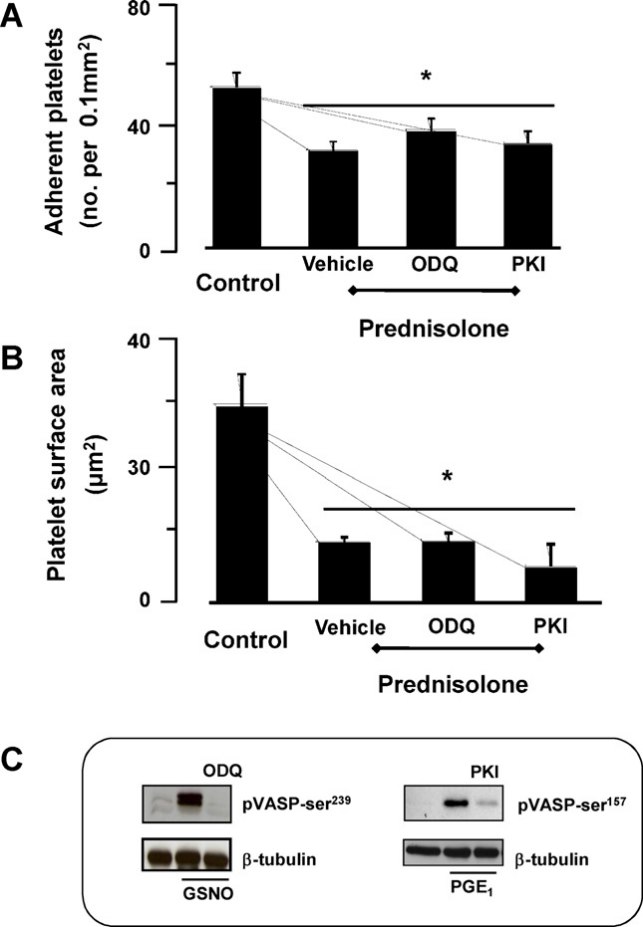
Role of Pred on the cGMP-signaling pathway. Glass slides were coated overnight at 4 °C with collagen (100 μg/ml), or heat-denatured humans serum. Uncoated surfaces were blocked by overlay with heat inactivated human serum albumin (HSA, 5%) for 30 min at 20 °C. Washed platelets (5 × 10^7^ platelets/ml) were pre-incubated with Pred (5 min; 10 μM) in the presence and absence of either ODQ (20 μM) or myrPKI (500 nM), and then adhered for 30 min, stained with TRITC-phalloidin and viewed using the 60× magnification. Platelets from 8 random visual fields with a total area of 0.1 mm^2^ were counted and expressed as number of adherent platelets/0.1 mm^2^ (A) and platelet surface area (μm^2^) (B), all data shown are mean ± SEM, *n* = 4 with separate blood donors. *P < 0.05 compared to collagen alone. (C) Washed platelets were pre-treated with either ODQ (20 mM) or myrPKI (500 nM) for 10 min and then incubated with either the nitric oxide donor, S-nitrosoglutathione (GSNO; 10 mM) or prostaglandin E_1_ (1 mM) for 2 min. Platelets were lysed and immunoblotted with either anti-VASP-phospho^239^ or anti-VASP-phospho^157^ antibodies.

Previously, we and others have shown that cAMP and cGMP signaling pathways reduce the release and activity of platelet ADP and TxA_2_ under both stirred and static conditions [16]. To explore this as a potential route of action for Pred, platelets were pre-incubated with the soluble guanylyl cyclase inhibitor, ODQ (20 μM) or the protein kinase A inhibitor myristolated PKI (500 nM). Under these conditions, Pred continued to inhibition platelet adhesion to collagen (Fig. 5A and B). These inhibitors blocked both nitric oxide and prostaglandin E1 induced phosphorylation of VASP on serine239 and 157, markers of protein kinase G and A respectively, confirming their activity under the conditions we employed (Fig. 5C). Supplementary Fig. 2 indicates that it is unlikely Pred could affect cAMP levels.

### 3.5 Platelet aggregation under conditions of flow

**Fig. 6:**
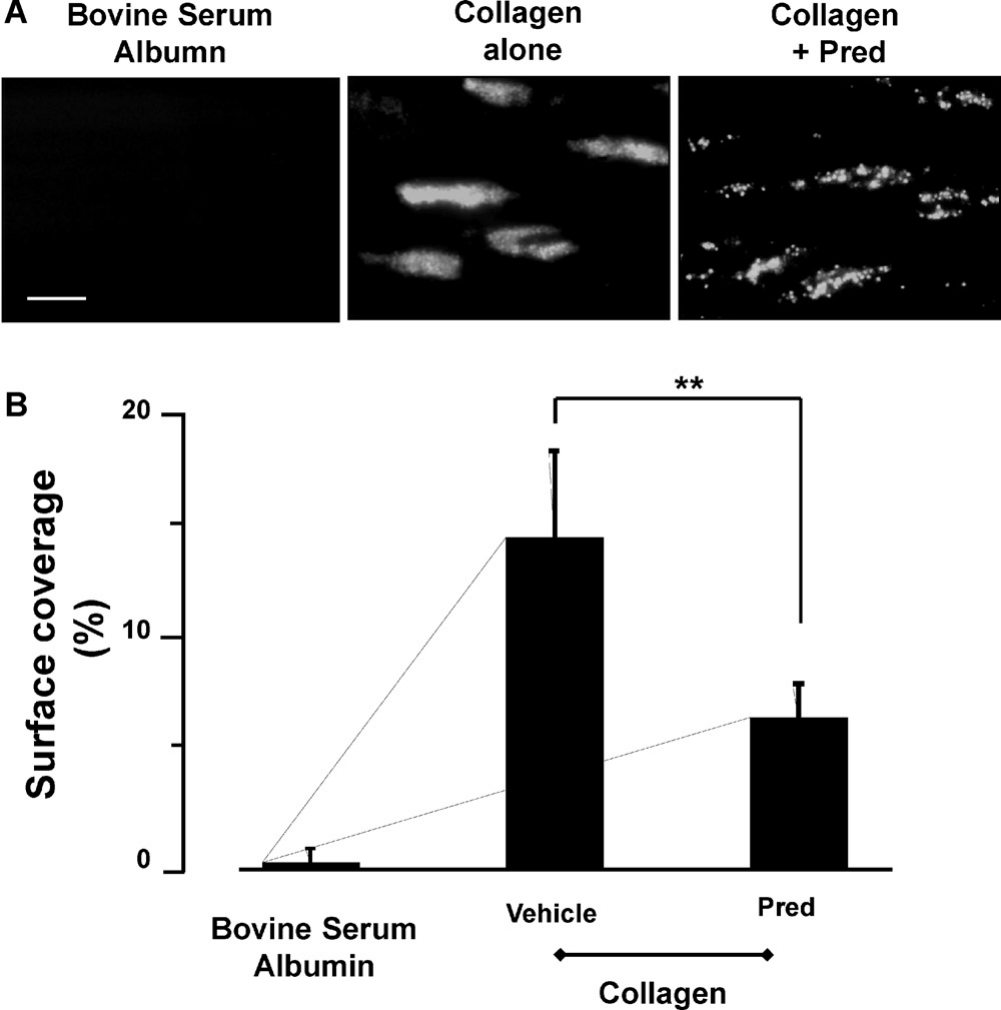
Prednisolone inhibits thrombus formation on collagen *in vitro*. Washed platelets were stained with DiOC_6_ (1 μM), incubated with Pred (10 μM) or vehicle for 2 min and reconstituted blood was flowed over immobilized collagen (100 μg/ml) for 4 min at a indicated shear rate of 1000/s^−1^. Platelet deposition viewed by fluorescence microscopy [21]. Images from 8 random fields of view were captured under fluorescence using cell^P imaging software (Olympus, Japan). Magnification, 60×. (A) Representative images. Bar, 20 μm. (B) Data are shown as percentage area coverage and are mean ± SD of *n* = 5 with separate blood donors. *P < 0.01 compared to collagen alone.

Finally, we examined the ability of Pred to regulate thrombus formation in response to collagen under shear conditions using an *in vitro* flow based assay. Under shear (1000 s^−1^), immobilized collagen (100 μg/ml) supported adhesion of numerous small thrombi that covered 14.7 ± 1.8% of the collagen-coated surface (Fig. 6). Pretreatment of platelets with Pred (10 μM) reduced surface coverage of collagen-coated microslides to 6.7 ± 1.1% (*P* < 0.01).

## 4 Discussion

There is mounting evidence that GC can also exert biological effects through non-genomic modalities. Recently we demonstrated that the synthetic GC Pred modulated platelet aggregation, through a mechanisms that involved engagement of the classical GR [11]. Since both platelets and GCs play important roles in the inflammation response, their interactions may have an important impact on a number disease states. The aim of the present study was to extend our initial observations to whole blood, a more physiologically relevant environment, and determine if this GC affected broad aspects of platelet function. We report that Pred, but not other GC, is active in the whole blood milieu and modulates both platelet-platelet and platelet–monocyte interactions. Furthermore, new data demonstrate that Pred reduces platelet adhesion and spreading, potentially through targeting the activation-dependent component of platelet adhesion to collagen. Importantly the downstream consequence of these actions, was that Pred could modulate thrombus formation *in vitro* under conditions of flow.

Our first critical observation was that Pred regulated platelet activity in whole blood, suggesting that the agent could be active against platelets in the circulation, a finding with obvious clinical bearing. Using the whole blood protocol, Pred induced a significant inhibition of ADP-induced aggregation, but was ineffective when collagen and AA were used to stimulate aggregation. These data are in part consistent with observations conducted with PRP and the aggregation assay; in the whole blood protocol Pred afforded only a modest effect on collagen-induced aggregation, while it affected collagen-induced responses in PRP [11]. We explain this apparent discrepancy because collagen can also stimulate neutrophils in the whole blood protocol [17], an effect that could mask inhibition of platelet aggregation. In fact, lower concentrations of collagen and AA may have inhibited aggregation to a significant extend, however the concentrations selected were the ones providing more reliable and consistent results in whole blood settings. Our new analyses showed that Pred inhibition of aggregation was maintained over time, since results obtained after 3 min were comparable to what observed following 1 h pre-incubation. Interestingly, Pred was unique in this ability [18], since the other GC tested failed to have any effect. We examined a number of different synthetic steroids, selected because of their differing affinities for GR [19]. Pred and fludrocortisone have shown an higher affinity for GR, but can also bind the mineralocorticoid receptor (MR) [20,21], Dex binds strictly bind GR [20], while triamcinolone [22] appears to be similar to Dex but with low affinity for the MR. In any event, Pred remained the only GC able to engage platelet GR to elicit non-genomic inhibitory responses. The NO-donating GC [23], Nitro-Dex, did inhibit aggregation, but this effect is clearly due to the NO moiety, which releases NO in biological fluids, and not to the GC moiety since Dex alone was ineffective. GR is a peculiar receptor, since it acts as a complex of which the ligand is fully part of [3,24]; we postulate that the side groups attached to the GC core structure of Pred are able to activate platelet GR in a specific and selective manner, though the structural motifs and reasons behind this would be the scope for future studies. Similar stringent structural motif can be postulated for Pred interaction (and activation thereof) with MR, since fludrocortisone was inactive in these settings. Altogether, this remains an intriguing set of data that might require thorough molecular pharmacology investigations to be clarified.

An increase of heterotypic leukocyte-platelet aggregates has been reported cardiovascular disease [25,26], which may contribute to their inflammatory component(s). Furthermore, reduction of the formation of these heterocellular aggregates is attained by widely used anti-platelet therapies such those presugrel or clopidogrel [27]. We found that, consistent with clinically used anti-platelet agents, Pred was a highly effective inhibitor of ADP-induced platelet/monocyte aggregate in whole blood. In addition, we could unveil a preferential effect on platelet, and not monocyte, reactivity as demonstrated by the re-constitution experiments. We conclude that platelet GR may be endowed with ‘higher modulatory’ function in the control of aggregate formation, a feature that is currently being extended to inflammatory pathologies too [26].

At sites of vascular injury platelet accumulation begins with an interaction between the extracellular matrix collagen and the platelet receptor glycoprotein VI [28]. This leads to activation of integrin α_2_β_1_ which then allow stable adhesion and facilitate spreading [29], a pre-requisite to thrombus formation [30]. Pred was extremely effective in inhibiting this fundamental platelet response, as measured by the extent of adhesion and spreading of platelets on collagen. A proportion of the adhesion response to collagen was resistant to Pred since complete inhibition was never attained regardless of the concentration applied. The effects of Pred on adhesion and spreading were lost under conditions where the effects of secondary signaling by ADP and TxA_2_ were abrogated. These data suggest strongly that Pred targets secondary signaling required for maximal platelet adhesion (hence the lack of complete inhibition) and is consistent with our observation that Pred preferentially targets ADP-induced aggregation and limit TxA_2_ synthesis [11].

The argument is further strengthen by aggregation data using combinations of Pred with known ADP receptor antagonists MRS2395 and A3P5P. In the presence of the P2Y_12_ inhibitor A3P5P, the ability of Pred to inhibit platelet was lost, while with combination of MRS2395, a P2Y_1_ antagonist, we observed an additive effect of Pred, strongly suggesting that this GC regulates AD-induced platelet activation through modulation of the P2Y_12_/Gi signaling pathway. We also found that in whole blood, Pred did not inhibit aggregation induced by either AA or collagen, which is in contrast to the data obtained using PRP. It is possible that the effects of Pred could be masked here due to the high concentrations of agonist required in whole blood assays, negating the reliance on secreted ADP. The influence of Pred on the complex relationship between secreted ADP and TxA_2_ is an area that requires further clarification.

Previously we have observed that cyclic nucleotide pathways, particularly those activated through NO, also act through a similar mechanism and adhesion is regulated through reduced TxA_2_ signaling and ADP bioavailability [14]. However, while Pred and NO seemed to act in a similar manner, Pred did not exert its effects through these pathways as evidenced by both its inability to increase cAMP/cGMP concentrations and by the fact that inhibitory actions were maintained under experimental conditions where cyclic nucleotide signaling was blocked. In addition, we measured NO species in the plasma of whole blood incubated with Pred and aggregants, and found no modulation (data not shown).

The adhesion and spreading of platelets on collagen are a prerequisite for subsequent thrombus formation, which is also known to require secondary signaling through ADP [13]. We reasoned that since Pred was such an effective inhibitor of spreading, it may also modulate thrombus formation. Our data show – for the first time – that Pred can exert dramatic effects on accumulation of platelets under flow. Indeed the effects of Pred were arguably more potent under flow than static conditions. This may reflect the transient nature of the interactions between platelets and collagen under flow, where signaling events take place in “spikes” rather than the *sustained signaling*, which takes places under static conditions. Thus, the inhibitory actions of Pred, may not be potent when competing with sustained activatory signaling, but under flow its inhibitory actions are magnified as it is only competing with transient signaling, enabling Pred to have a profound effect on thrombus formation.

In conclusion, Pred can inhibit platelet adhesion, spreading, aggregation, thrombus formation, and their interaction with monocytes, through a novel mechanism that targets secondary signaling. Initial evidence seems to point to the P2Y_12_ providing guidance for our follow up studies. In any case, our current observations are highly relevant to cardiovascular and inflammatory clinical settings and set the basis for further investigation aiming to detail Pred mechanism of action. The last point could be particularly true in view of the fact that Pred is the most widely used GC in the clinical environment.

## Figures and Tables

**Table 1:** Stimulus-dependency for Pred inhibition of platelet aggregation in whole blood.

Agonist	ADP (μM)			Arachidonic acid (μM)		Collagen
	6.50	3.22	1.56	250	500	
Inhibition (%)	22.7 ± 5.6*	19.2 ± 4.0*	2.0 ± 1.0	4.0 ± 1.0	2.0 ± 1.0	9.5 ± 2.0

Platelets were incubated with 5 μM prednisolone for 3 min prior to stimuli addition (collagen was used at 3.2 μg/ml) and aggregation monitored as described in Section 2. Values (mean ± SEM) report the percentage of inhibition as calculated for each stimulus/concentration out of 5 distinct experiments.

* *P* < 0.05 as calculated on the original values.

## References

[1] Adcock I.M. (2000). Molecular mechanisms of glucocorticosteroid actions. Pulm Pharmacol Ther.

[2] Liberman A.C., Druker J., Garcia F.A., Holsboer F., Arzt E. (2009). Intracellular molecular signaling basis for specificity to glucocorticoid anti-inflammatory actions. Ann N Y Acad Sci.

[3] Buttgereit F., Scheffold A. (2002). Rapid glucocorticoid effects on immune cells. Steroids.

[4] Schulz M., Schneider S., Lottspeich F., Renkawitz R., Eggert M. (2001). Identification of nucleolin as a glucocorticoid receptor interacting protein. Biochem Biophys Res Commun.

[5] Lowenberg M., Verhaar A.P., van den Brink G.R., Hommes D.W. (2007). Glucocorticoid signaling: a nongenomic mechanism for T-cell immunosuppression. Trends Mol Med.

[6] Bartholome B., Spies C.M., Gaber T., Schuchmann S., Berki T., Kunkel D. (2004). Membrane glucocorticoid receptors (mGCR) are expressed in normal human peripheral blood mononuclear cells and up-regulated after in vitro stimulation and in patients with rheumatoid arthritis. Faseb J.

[7] Lowenberg M., Verhaar A.P., Bilderbeek J., Marle J., Buttgereit F., Peppelenbosch M.P. (2006). Glucocorticoids cause rapid dissociation of a T-cell-receptor-associated protein complex containing LCK and FYN. EMBO Rep.

[8] Lowenberg M., Tuynman J., Bilderbeek J., Gaber T., Buttgereit F., van Deventer S. (2005). Rapid immunosuppressive effects of glucocorticoids mediated through Lck and Fyn. Blood.

[9] Yazid S., Leoni G., Getting S.J., Cooper D., Solito E., Perretti M. (2010). Antiallergic cromones inhibit neutrophil recruitment onto vascular endothelium via annexin-A1 mobilization. Arterioscler Thromb Vasc Biol.

[10] Bishop-Bailey D. (2010). The platelet as a model system for the acute actions of nuclear receptors. Steroids.

[11] Moraes L.A., Paul-Clark M.J., Rickman A., Flower R.J., Goulding N.J., Perretti M. (2005). Ligand-specific glucocorticoid receptor activation in human platelets. Blood.

[12] Schafer A., Bauersachs J. (2008). Endothelial dysfunction, impaired endogenous platelet inhibition and platelet activation in diabetes and atherosclerosis. Curr Vasc Pharmacol.

[13] Roberts W., Riba R., Homer-Vanniasinkam S., Farndale R.W., Naseem K.M. (2008). Nitric oxide specifically inhibits integrin-mediated platelet adhesion and spreading on collagen. J Thromb Haemost.

[14] Roberts W., Michno A., Aburima A., Naseem K.M. (2009). Nitric oxide inhibits von Willebrand factor-mediated platelet adhesion and spreading through regulation of integrin alpha(IIb)beta(3) and myosin light chain. J Thromb Haemost.

[15] Shattil S.J., Kashiwagi H., Pampori N. (1998). Integrin signaling: the platelet paradigm. Blood.

[16] Davi G., Patrono C. (2007). Platelet activation and atherothrombosis. N Engl J Med.

[17] Garnotel R., Wegrowski J., Bellon G., Monboisse J.C., Perreau C., Borel J.P. (1993). Adhesion and activation of human neutrophils onto collagen chains separated by electrophoresis. Exp Cell Res.

[18] Siller-Matula J.M., Christ G., Lang I.M., Delle-Karth G., Huber K., Jilma B. (2010). Multiple electrode aggregometry predicts stent thrombosis better than the vasodilator-stimulated phosphoprotein phosphorylation assay. J Thromb Haemost.

[19] Rupprecht R., Reul J.M., van Steensel B., Spengler D., Soder M., Berning B. (1993). Pharmacological and functional characterization of human mineralocorticoid and glucocorticoid receptor ligands. Eur J Pharmacol.

[20] Grossmann C., Scholz T., Rochel M., Bumke-Vogt C., Oelkers W., Pfeiffer A.F. (2004). Transactivation via the human glucocorticoid and mineralocorticoid receptor by therapeutically used steroids in CV-1 cells: a comparison of their glucocorticoid and mineralocorticoid properties. Eur J Endocrinol.

[21] Igarashi Y. (1994). [Synthetic mineralocorticoid, clinical application of fludrocortisone acetate (Florinef)]. Nippon Rinsho.

[22] Weyts F.A. (1998). Verburg-van Kemenade BM, Flik G. Characterisation of glucocorticoid receptors in peripheral blood leukocytes of Carp, Cyprinus carpio L. Gen Comp Endocrinol.

[23] Perretti M., Paul-Clark M.J., Mancini L., Flower R.J. (2003). Generation of innovative anti-inflammatory and anti-arthritic glucocorticoid derivatives that release NO: the nitro-steroids. Dig Liver Dis.

[24] Miner J.N., Hong M.H., Negro-Vilar A. (2005). New and improved glucocorticoid receptor ligands. Expert Opin Investig Drugs.

[25] Gremmel T., Kopp C.W., Seidinger D., Giurgea G.A., Koppensteiner R., Steiner S. (2009). The formation of monocyte-platelet aggregates is independent of on-treatment residual agonists’-inducible platelet reactivity. Atherosclerosis.

[26] Michelson A.D., Barnard M.R., Krueger L.A., Valeri C.R., Furman M.I. (2001). Circulating monocyte-platelet aggregates are a more sensitive marker of in vivo platelet activation than platelet surface P-selectin: studies in baboons, human coronary intervention, and human acute myocardial infarction. Circulation.

[27] Braun O.O., Johnell M., Varenhorst C., James S., Brandt J.T., Jakubowski J.A. (2008). Greater reduction of platelet activation markers and platelet–monocyte aggregates by prasugrel compared to clopidogrel in stable coronary artery disease. Thromb Haemost.

[28] Watson S.P., Gibbins J. (1998). Collagen receptor signalling in platelets: extending the role of the ITAM. Immunol Today.

[29] Behnke O. (1970). Microtubules in disk-shaped blood cells. Int Rev Exp Pathol.

[30] Born G.V. (1970). Observations on the change in shape of blood platelets brought about by adenosine diphosphate. J Physiol.

