# Supplementary material for: Prednisolone exerts exquisite inhibitory properties on platelet functions
Source: Biochem Pharmacol. 2012 May 15;83(10):1364–73. doi: 10.1016/j.bcp.2012.02.006 (PMC3320711; doi:10.1016/j.bcp.2012.02.006)
Supplement: Supplementary file 1 [file mmc1.ppt]

## Slide 1
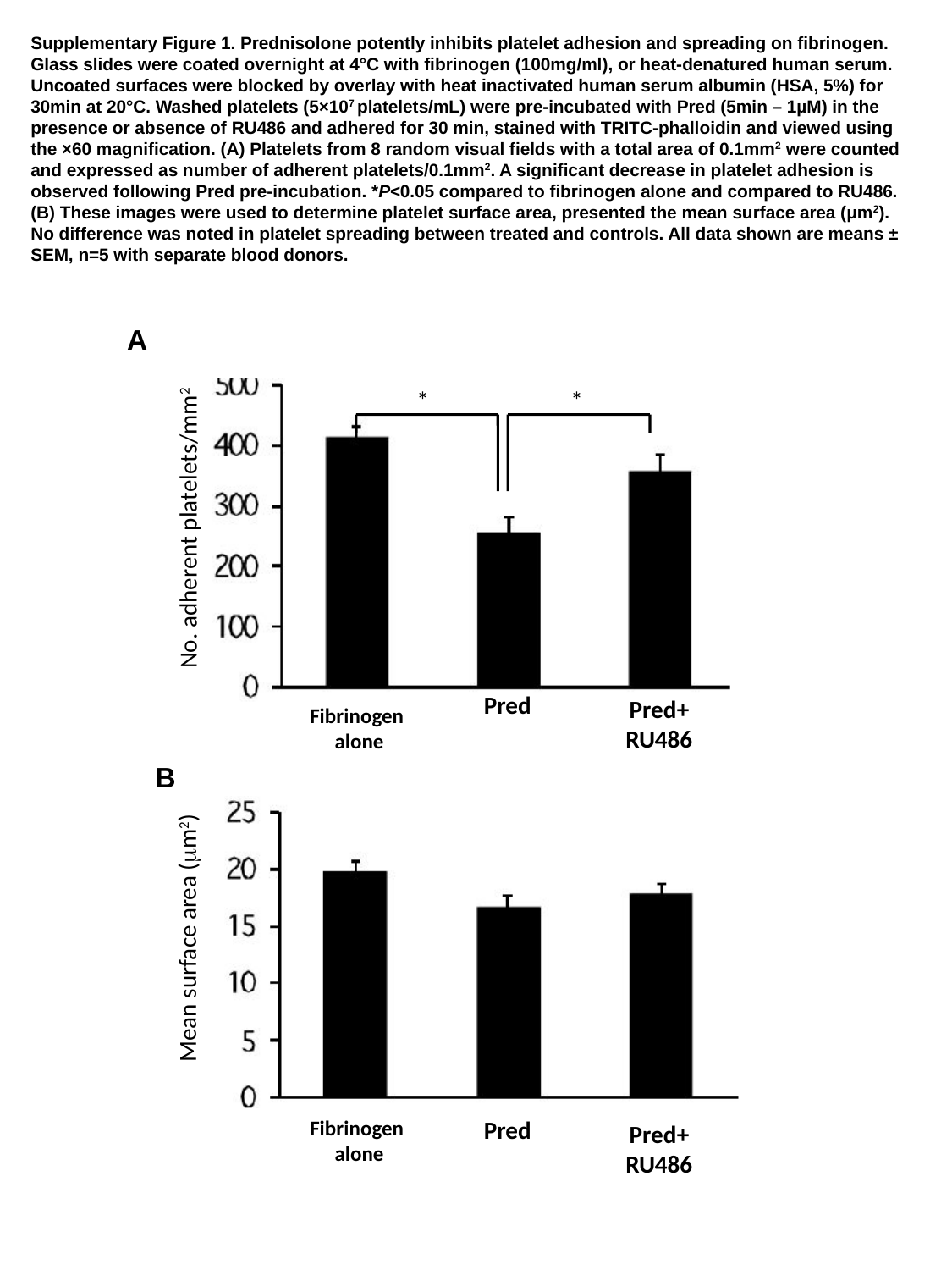

Supplementary Figure 1. Prednisolone potently inhibits platelet adhesion and spreading on fibrinogen. Glass slides were coated overnight at 4°C with fibrinogen (100mg/ml), or heat-denatured human serum. Uncoated surfaces were blocked by overlay with heat inactivated human serum albumin (HSA, 5%) for 30min at 20°C. Washed platelets (5×10^7^ platelets/mL) were pre-incubated with Pred (5min – 1µM) in the presence or absence of RU486 and adhered for 30 min, stained with TRITC-phalloidin and viewed using the ×60 magnification. (A) Platelets from 8 random visual fields with a total area of 0.1mm2 were counted and expressed as number of adherent platelets/0.1mm2. A significant decrease in platelet adhesion is observed following Pred pre-incubation. *P<0.05 compared to fibrinogen alone and compared to RU486. (B) These images were used to determine platelet surface area, presented the mean surface area (μm2). No difference was noted in platelet spreading between treated and controls. All data shown are means ± SEM, n=5 with separate blood donors.
A
*
*
No. adherent platelets/mm2
Pred
Pred+
RU486
Fibrinogen
 alone
B
Mean surface area (μm2)
Fibrinogen
 alone
Pred
Pred+
RU486

## Slide 2
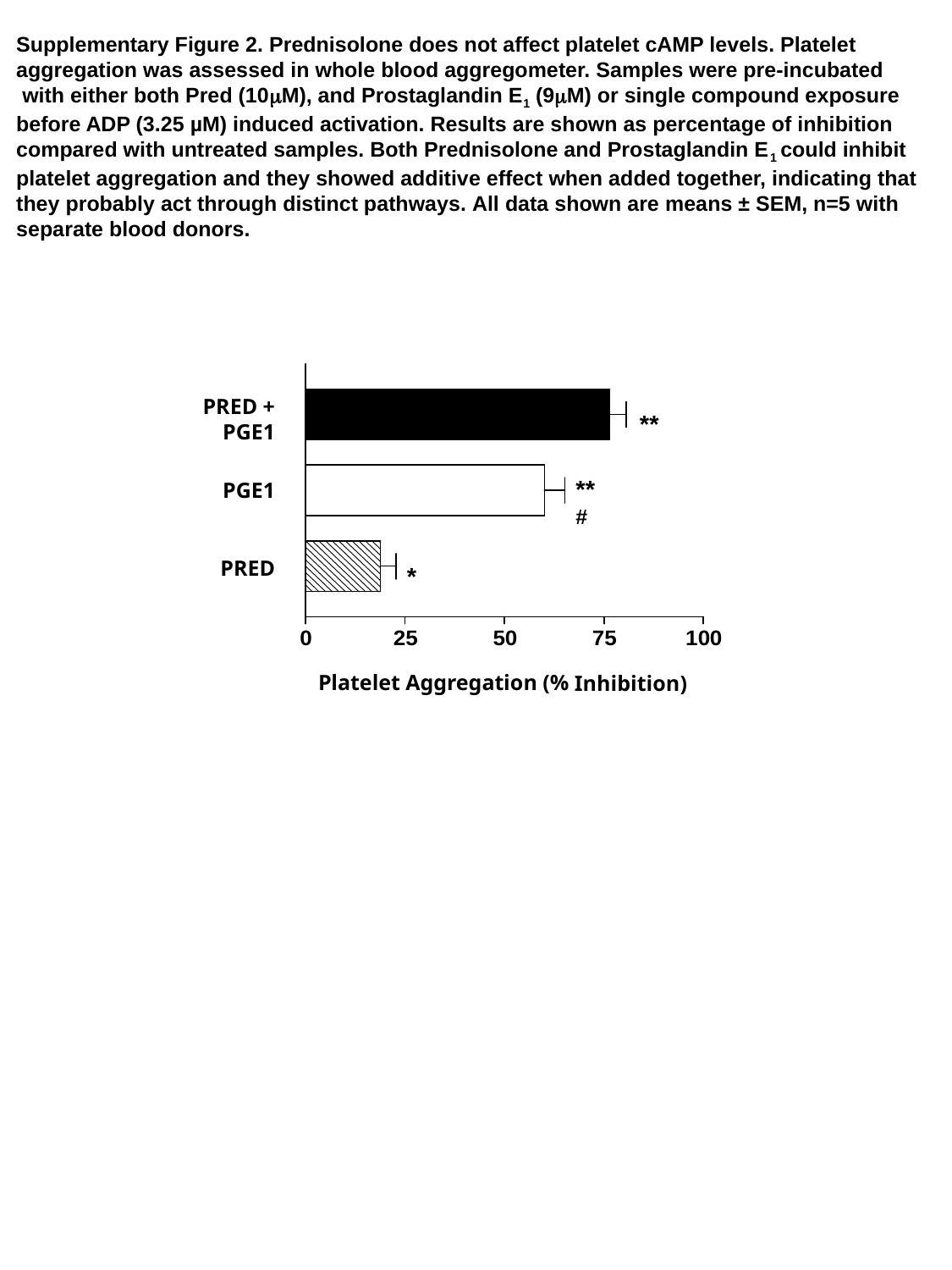

Supplementary Figure 2. Prednisolone does not affect platelet cAMP levels. Platelet aggregation was assessed in whole blood aggregometer. Samples were pre-incubated
 with either both Pred (10μM), and Prostaglandin E1 (9μM) or single compound exposure before ADP (3.25 µM) induced activation. Results are shown as percentage of inhibition compared with untreated samples. Both Prednisolone and Prostaglandin E1 could inhibit platelet aggregation and they showed additive effect when added together, indicating that they probably act through distinct pathways. All data shown are means ± SEM, n=5 with separate blood donors.
PRED + PGE1
**
**
#
PGE1
PRED
*
Platelet Aggregation (% Inhibition)
